# Statistical-Based Optimization of Modified *Mangifera indica* Fruit Starch as Substituent for Pharmaceutical Tableting Excipient

**DOI:** 10.3390/polym16182653

**Published:** 2024-09-20

**Authors:** Prin Chaksmithanont, Ketsana Bangsitthideth, Kwanputtha Arunprasert, Prasopchai Patrojanasophon, Chaiyakarn Pornpitchanarong

**Affiliations:** 1 Department of Industrial Pharmacy, Faculty of Pharmacy, Silpakorn University, Nakhon Pathom 73000, Thailand; 2 Health Intervention and Technology Assessment Program (HITAP), Ministry of Public Health, Nonthaburi 11000, Thailand; 3 Research and Innovation Center for Advanced Therapy Medicinal Products, Faculty of Pharmacy, Silpakorn University, Nakhon Pathom 73000, Thailand

**Keywords:** mango, modified starch, pre-gelatinization, hydrolysis, tablet excipient

## Abstract

This study aimed to optimize modified starch from *Mangifera indica* (mango) fruit using acid hydrolysis and pre-gelatinization via computer-assisted techniques as a substituent for pharmaceutical tableting excipients. The hydrolysis and microwave-assisted pre-gelatinization time and temperature were optimized using a three-level factorial design. The modified starches were characterized for flowability, compressibility, and swelling properties. It was found that all parameters fit a quadratic model, which can be used to predict the properties of the modified starch. The optimized hydrolysis reaction was 3.8 h at 56.4 °C, while the pre-gelatinization reaction was 3 min at 150 °C. Structural changes were found, ascertaining that starch modification was successful. The optimized hydrolyzed starch showed superior properties in relative to unmodified M. indica fruit starch and comparable characteristics to conventional excipients. The optimized pre-gelatinized starch presented an excellent enhancement in the flow and compression properties, with %swelling greatly augmented 3.95-fold and 1.24-fold compared to unmodified starch and SSG, respectively. Additionally, the pre-gelatinized starch presented comparable binding effect, while the hydrolyzed powder had reduced binding capacity due to shorter chains. The findings revealed that the use of software-assisted design of experiment facilitated a data-driven approach to optimize the modifications. The optimized modified mango starch demonstrated potential as a multifunctional excipient, capable of functioning as binder, disintegrant, and diluent.

## 1. Introduction

Pharmaceutical tablet excipients are inactive substances used as carriers to allow the formulation, manufacturing, and delivery of the active ingredients. Excipients play a crucial role in ensuring the stability, bioavailability, and manufacturability of the final product [1]. Traditional excipients, such as microcrystalline cellulose, lactose, and various starches, have been widely used due to their proven efficacy and safety profiles [2]. Specifically, different forms of starch play versatile and essential roles in tablet formulations by functioning as binders, disintegrants, fillers, glidants, controlled release agents, stabilizers, thickeners, and film-forming agents [3]. These contribute to the overall quality, performance, and efficacy of pharmaceutical tablets [4].

In recent years, the increasing demand for more sustainable, biocompatible, and cost-effective alternatives has spurred interest in exploring novel, naturally derived excipients [5,6]. The pharmaceutical industry has faced significant challenges, including the need for greener production processes and the growing preference for natural and organic products among consumers [7]. The need for greener production processes was considered due to regulatory and consumer demands as well as the global sustainability trend [8,9]. The pharmaceutical industry is increasing the adaptation of natural sources of tableting excipients to address consumer demand for safer, cleaner, and more environmentally sustainable products [10]. The acquisition of natural excipients offers functional, economical, and environmental benefits. The production of newer excipients, potato, maize, and pinhão starch emerges as an approach for chemical excipient replacement [4,11]. Moreover, the use of phytopharmaceutical is another example of a green medicinal production through the CO_2_-based energy expenditures instead of active ingredient chemical syntheses through organic solvents [12]. The naturally-derived alternatives promise not only to meet the functional requirements of conventional excipients but also to offer additional benefits, such as enhanced biodegradability, reduced environmental impact, and the potential for improved patient compliance due to their natural origins [13]. Several naturally derived starches have been explored as tableting excipients—for example, banana, cassava, tapioca, mango, etc. [14,15,16,17].

Mango (*Mangifera indica*) is a tropical plant mostly found in India and Southeast Asia. The mango fruit is harvested and traded for consumption worldwide [18]. One of the major components in mango fruit, especially when unripe, is starch, which makes the fruit a promising candidate as a tableting excipient [19]. Former reports have shown that mango-derived starch showed good swelling capacity and flowability, making it suitable for various pharmaceutical applications [20,21]. Thus, the modification of mango starch would enrich its potential to a specific function and may allow the naturally derived starch to replace synthetic excipients. Furthermore, the use of mango starch can contribute to the valorization of agricultural by-products, reducing waste and promoting circular economy [22,23]. The use of mango-derived starch presented as an alternative to potato and maize for starch production. Mango, especially when unripe, contains high amounts of starch and can serve as an alternative starch source. In mango-growing regions, the cost of producing starch from mango could be lower than producing starch from maize or potatoes, which require significant agricultural inputs. Moreover, mango may serve as a more sustainable source of starch as it grows with abundant sunlight and minimal input requirements of the tropical region, compared to the more resource-intensive production of potatoes and maize which are temperate climate crops [24,25]. This adds value to mango farming, making it more sustainable than starches derived from crops that require dedicated cultivation. Therefore, the development of modified mango fruit starch is a crucial step in empowering the efficacy of the tropical fruit in the pharmaceutical industry.

To enhance the functional properties of mango starch and broaden its application as a tableting excipient, various modification techniques can be employed [26,27]. Among these, acid hydrolysis and pre-gelatinization are two notable methods that have shown significant possibilities. Acid hydrolysis involves the partial breakdown of starch into shorter polysaccharide chains using acid. This process typically involves treating the starch with a dilute acid solution at controlled time and temperature [28]. This reaction reduces the molecular weight of starch and modifies its crystalline structure, resulting in improved solubility, reduced viscosity, and enhanced flow properties, which are advantageous for tablet formulation [29]. Moreover, pre-gelatinization involves the gelatinization of starch granules followed by drying, resulting in a modified starch that has undergone both physical and chemical changes [30]. The process would yield a modified starch with enhanced water absorption capability which may lead to improved swelling capacity and binding properties. Additionally, pre-gelatinized starch exhibits superior compressibility, which is beneficial for tablets manufacturing process, particularly in direct compression tableting [31]. Thus, the modification of mango starch through acid hydrolysis and pregelatinization could significantly enhance its suitability as a tableting excipient, offering versatility and improved performance in pharmaceutical applications [31].

This work aimed to develop modified *M. indica* fruit starch via computer software-assisted optimization to deliver optimized modified starch suitable as substituent for pharmaceutical tableting excipient. Starch was extracted from the *M. indica* fruit and modified using acid-hydrolysis and pre-gelatinization processes. Furthermore, the characteristics and attributes of the modified starch was studied in comparison to the conventional excipients to prove their potential to replace excipient functions, including diluent, disintegrant, and binder. The use of software-assisted design of experiment (DOE) provides a data-driven framework for making decisions. The analysis of experimental data through statistical methods would ensure that conclusions are based on objective evidence, leading to more reliable modification of *M. indica* fruit starch.

## 2. Materials and Methods

### 2.1. Materials

*Mangifera indica* was purchased from different local markets in Nakhon Pathom, Thailand. Microcrystalline cellulose (MCC) eq. AVICEL^®^ PH102, sodium starch glycolate (SSG), and povidone K30 (PVP K30) were bought from P.C. Drug Center (Bangkok, Thailand). Other chemicals and solvents were used as received.

### 2.2. Preparation of M. indica Fruit Starch

*M. indica* fruit was thoroughly washed to remove dirt and soil. The cleaned epicarp (outer skin) of *M. indica* fruit was peeled off and the seed was removed. The mesocarp (pulp) of the fruit was sliced and dried in a hot air oven at 50 °C until a constant weight was achieved. The dried *M. indica* was then chopped, ground, and sieved through a 100-mesh sieve to produce *M. indica* flour.

To extract starch from the flour, 30 g of *M. indica* flour was mixed with 100 mL of purified water and homogenized for 30 min. After extraction, the mixture was left to separate for at least 6 h. The dispersion liquid was discarded, leaving solid sediment. The sediment was washed twice with excess purified water and filtered through Whatman filter paper No. 1. The starch was collected after drying in a hot air oven at 50 °C.

### 2.3. Optimization of Hydrolyzed M. indica Starch

Acid hydrolysis was used to prepare hydrolyzed *M. indica* starch [32]. The optimal time and temperature used to perform the reaction yielding a desirable starch as tableting excipient was optimized predicted using experimental design with computer-software assistance (Design-Expert^®^ 11, Stat-Ease, Minneapolis, MN, USA). The three-level factorial response-surface design was used to vary the time (1–6 h, A) and temperature (40–60 h, B). The coded and actual range of each independent variable are presented in Table 1. The impact of these parameters on the dependent variables—Hausner ratio, Carr index, and %swelling—was investigated after the generation of experimental runs by the computer software. The response values were analyzed using ANOVA in the Design-Expert^®^ 11 software to examine the interaction effects between two variables and to determine the optimal level for each factor.

Briefly, 30 g of *M. indica* fruit starch in 100 mL of 3.4% HCl solution was prepared and stirred at various times and temperatures according to the design of experiments. After that, a 5 N NaOH solution was used to adjust the pH to 5.5. The suspension was centrifuged at 4000 rpm for 10 min. Then, the precipitates were further dried in a hot air oven at 50 °C. The dried hydrolyzed starch was grinded through sieve mesh No. 100 and stored in a desiccator until use.

### 2.4. Optimization of Pre-Gelatinized M. indica Starch

The pre-gelatinization reaction was conducted using microwave-assisted approach. The time (1–5 min, A) and temperature (100–150 h, B) were varied via a 3-level factorial response–surface design by Design-Expert^®^ 11, software in which 14 experimental runs were suggested. The coded and actual range of each independent variable are presented in Table 2. The responses, Hausner ratio, Carr index, and %swelling—were investigated for the prediction of the optimized pre-gelatinized *M. indica* starch. The response surface methodology (RSM) was employed with a regression equation to identify the correlation between the independent and dependent variables. The results were analyzed by the software. The significance level was established at a 95% confidence interval with a *p*-value of 0.05.

To pre-gelatinize *M. indica* starch, 3 g of *M. indica* starch was dispersed in 25 mL of purified water and placed in a microwave glass vial [33]. Then, the microwave synthesizer (CEM Discover SP, Matthews, NC, USA) was used with the conditions generated by the design of experiment. After the predetermined reaction time, the microwave glass vial was settled in an ice bath to terminate the reaction. Then, the mixtures were transferred to a centrifuge tube and precipitated at 4000 rpm for 10 min. The supernatant was discarded, and the solid was dry at 50 °C in a hot air oven. The dried pre-gelatinized starch was pulverized and sieved through mesh No. 100 and desiccated until use.

### 2.5. Characterizations of Modified M. indica Starch

#### 2.5.1. Flowability and Compressibility

The flowability, presented by Hausner ratio, and the Carr’s compressibility index (Carr index) can predict powder flow characteristics, which are influenced by factors such as particle size and shape, material density, surface area, moisture content, and powder cohesiveness. These indices are determined using the untapped and tapped bulk density or the untapped and tapped bulk volume of a powder. Each starch sample was weighed (5 g) and placed into a 10-mL cylinder. The volume of the starch was collected before tapping as bulk volume (V_0_). Then, the cylinder was tapped 300 times from the same height such that no further volume changes occurred. The volume after tapping (tapped volume, V_f_) was measured. The commercial SSG, MCC, and PVP K30 were used as controls. The Hausner ratio and Carr index were calculated using Equation (1) and Equation (2), respectively.

(1) $$Hausner\;ratio=\frac{V_{0}}{V_{f}}$$

(2) $$Carr\;index=\frac{V_{0}-V_{f}}{V_{o}}\times 100$$

#### 2.5.2. Swelling Index

The swelling property of the modified starch and controls was examined by adding 1 g of starch into a 10-mL cylinder and the volume of starch was collected (V_0_). Ten mL of purified water was added, then starch was allowed to swell for 24 h. The swollen volume (V_s_) was recorded and %swelling was calculated using Equation (3).

(3) $$\%Swelling=\frac{V_{s}-V_{0}}{V_{s}}\times 100$$

#### 2.5.3. Attenuated Total Reflectance Fourier-Transformed Infrared Spectroscopy

The molecular characteristics of the modified starch were collected using attenuated total reflectance Fourier-transformed infrared (ATR-FTIR) spectroscopy (Nicolet iS5, Thermo Fisher Scientific, Bedford, MA, USA). The starch samples were placed on the ATR diamond and the spectra (wavenumber range from 400–4000 cm^−1^) were collected at a resolution of 4 cm^−1^ and 16 running scan.

#### 2.5.4. Differential Scanning Calorimetry

Differential scanning calorimeter (DSC) (Sapphire, PerkinElmer, Waltham, MA, USA) was employed to analyze the thermal property of the modified *M. indica* starch. Each sample was weighed (5 mg) and placed in an aluminum pan, hermetically sealed, and heated to between 40–200 °C at a rate of 20 °C/min under constant nitrogen flow of 50 mL/min.

### 2.6. Modified M. indica Fruit Starch as Excipient Substituent in Acetaminophen Tablets

To demonstrate *M. indica* fruit starch function as binder, the starch was formulated in the acetaminophen tablet to examine the hardness and friability of the tablets compared to PVP K30. The tablets were formulated according to the formulae in Table 3 the using direct compression method. The drug and inactive ingredients were mixed and grinded through sieve mesh No. 100. Then, magnesium stearate was added prior to tableting. Each tablet weighed 0.440 g and was compressed into tablets using a hydraulic press (Manual Press, Specac, Orpington, UK) with compression force of 5 tons.

### 2.7. Performance Tests

#### 2.7.1. Hardness

Ten tablets were randomly sampled for hardness measurement using the tablet hardness tester (ERWEKA^®^ Tablet Hardness Tester TBH 325 Series, Langen, Germany). The data was collected in newton (N) force.

#### 2.7.2. Friability

The friability of the tablets was evaluated using a friability tester (ERWEKA^®^ TAR series, Langen, Germany) using the method described in the United States Pharmacopeia 2024 general chapter (1216) TABLET FRIABILITY. As each tablet weighed less than 0.65 g, the tablets were dedusted, sampled, and weighed as near as possible to 6.5 g. The accurately weighed tablet samples were placed in the drum and rotated 100 times at a speed of 25 rpm. Thereafter, the tablets were collected, any loose dust was removed, and they were accurately re-weighed (no cracked, cleaved, or broken tablets were presented). Weight loss from a single test or the mean of three tests of less than 1.0% of the initial weight was considered acceptable.

### 2.8. Statistical Analysis

All experiments were conducted in triplicates and reported as mean ± standard deviation. Statistical analysis was conducted using F-test and independent *t*-test to establish significant differences, when *p* < 0.05.

## 3. Results and Discussion

### 3.1. Preparation of M. indica Fruit Starch

The gathering of *M. indica* fruits used in this research was conducted to minimize the variation of starch content in the most possible approaches. The fruits were bought from different local markets in the same season within no longer than a 1-month timeframe. The epicarp was thoroughly cleansed and carefully peeled off to maintain the highest content of the mesocarp possible. The dried–ground mesocarp (*M. indica* fruit flour) yielded 57.15 ± 9.23% relative to the initial weight of the mesocarp. The amount of *M. indica* fruit flour was satisfactory since the amount of water in unripe mango fruit was reported to be approximately 25–30% [34]. The variations were expected due to numerous factors that may have influenced the amount of solid content in the fruit, e.g., source of the plant, season gathered, strains, ripen stage, etc.

Once the flour was acquired in the extraction of *M. indica* fruit starch, it was found that 40 ± 5.04% of starch was obtained from the mesocarp. This is in accordance with Lagunes-Delgado et al., (2022) which reported that 30–45% of starch can be found from unripen pulp of mango and starch is one of the major constituents in the mango fruit pulp closely to soluble sugars (30–34%, in dried basis) [35]. Therefore, the amount of extracted starch was desirable and used for further modifications.

### 3.2. Optimization of Hydrolyzed M. indica Fruit Starch

The optimization using computer-software assisted approach was conducted by varying the reaction time and temperature. The 3-level factorial response–surface design generated 14 experimental runs as shown in Table 3. The hydrolysis times (A) were 1, 3.5, and 6 h, while the 3 levels of reaction temperature (B) were 40, 50, and 60 °C. After each hydrolysis reaction and neutralization, the starch dispersion turned from pale yellow to brownish suspension and presented a unique mango scent. The three-level factorial design was employed to model potential curvature in the response function and to manage nominal factors set at three levels.

As presented in Table 4, the Hausner ratio representing flowability of the starch was between 1–2, the Carr index was in the range of 10–50, and the %swelling was 250–360%. The results indicated a wide range of flowability differences from excellent to very poor flow. The flowability of a powder can be assessed using compressibility or the Hausner ratio [36]. Better flow properties are indicated by lower compressibility or a lower Hausner ratio. A Hausner ratio of less than 1.11 signifies “excellent” flow, 1.12–1.18 shows good flow character, 1.19–1.25, 1.26–1.34, 1.35–1.45, and 1.46–1.59 designate fair, passable, poor, and very poor flow, respectively, while a Hausner ratio greater than 1.60 indicates “very very poor” flow. Starch with excellent flow property is ideal for direct compression and high-speed tablet manufacturing. The better flow property would minimize the risk of segregation, uniform die filling, and consistent tablet weight. On the contrary, starch with higher Hausner ratios may bring about problems in consistent and efficient production. In accordance with the flow property, the compressibility determined through Carr index also varied from excellent (0–10) to very very poor (>38) [37]. The Carr index provides a quantitative measure of compressibility or the ability to decrease in volume under pressure. Lower Carr index values indicate lower compressibility, making them easier to handle, process, and maintain at a more consistent volume, which leads to uniform tablet weight and size. Lastly, the swelling property of a powder is another important factor in tablet formulation and has numerous effects on the tableting process and the characteristics of the final tablet (disintegration, dissolution rate, tablet size and weight, etc.). A higher %swelling signifies a greater ability of the starch to absorb water and expand which would influence tablet disintegration and dissolution, thus enhancing the bioavailability of the active ingredient.

According to the summary of ANOVA and multiple regression analysis in Table 5, The *p*-values for all responses were less than 0.05, indicating that the quadratic model was able to describe the significant relationships between the dependent and all independent variables. The lack-of-fit *p*-value of more than 0.05 designated that the multiple regression model was appropriate. Additionally, the higher R^2^ values presented the reliability of the fitted mathematical model. Hence, the obtained R^2^ proved that the predicted models were suitable and accurately explain the relationship of the variables. Given the significant dependent variables, the coded equations reported can be used to predict Hausner ratio, Carr index, and %swelling of the hydrolyzed starch. These equations allowed the prediction of responses based on specific levels of each component. By comparing the coefficients of the factors, the relative importance of the components can be assessed using the coded equation.

**Figure 1:**
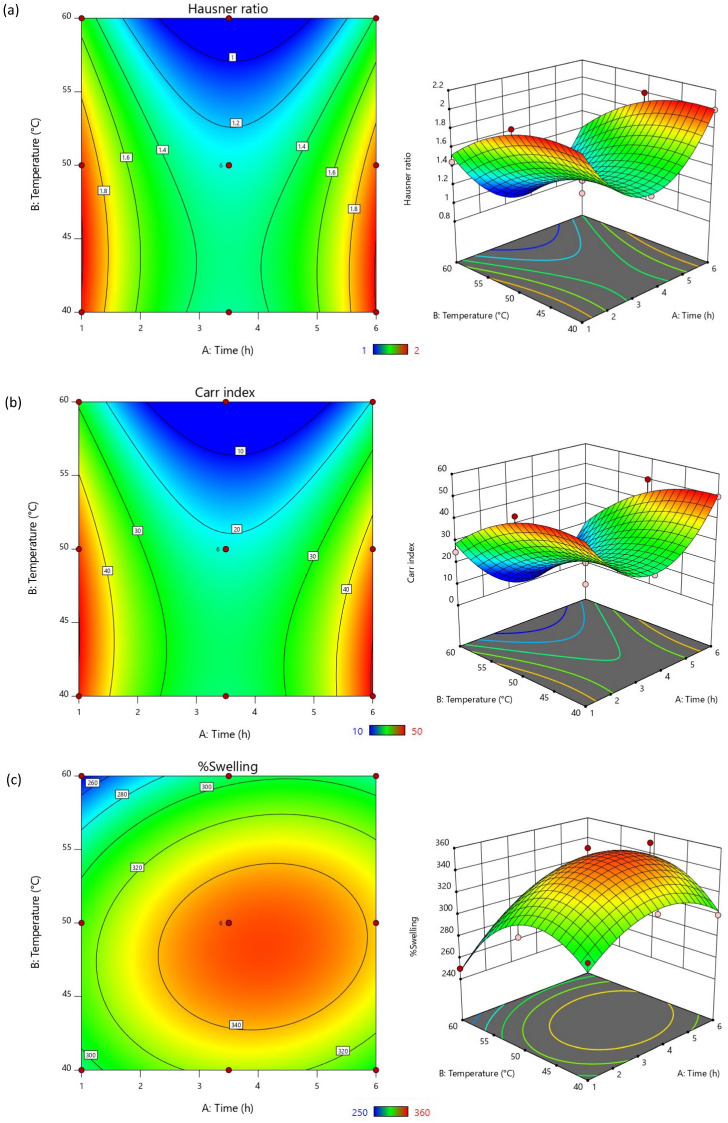
Two-dimensional contour plots and three-dimensional response surface graphs representing the effects of hydrolysis time and temperature on (**a**) Hausner ratio, (**b**) Carr index, and (**c**) %swelling.

The response surface graphs (Figure 1) can be used to describe the relationship and impact of the input factors on the output responses with the higher values were presented in red and the lower values are shown in blue areas, respectively. The hydrolysis time (A), temperature (B), and the interaction of these factors (AB) impacted negatively on the flow and compressibility characteristics of the obtained starch. This suggested that increasing the time and temperature of the reaction resulted in lower Hausner ratio and Carr index, which are preferrable [38]. Considering the %swelling, the results were negatively influenced by the reaction temperature, yet positively influenced by the reaction time and the interaction of the input factors. Acid hydrolysis breaks down the starch molecules into smaller fragments by cleaving the glycosidic bonds, leading to the reduction in molecular weight, lower viscosity, and flowability changes. The shorter starch chains may reduce the cohesive forces between starch particles. This phenomenon could have improved the flow properties and compressibility of the *M. indica* fruit starch. The longer acid hydrolysis time led to more extensive hydrolysis at the glycosidic bonds, producing lower molecular weight fragments, thereby reducing the cohesive forces. Together with the hydrolysis time, the increased reaction temperature could accelerate the reaction rate of chain cleavage leading to faster hydrolysis [39,40]. Although starch hydrolysis could also cause the modified starch to gelatinize by absorbing water and swell, the higher hydrolysis temperature was found to reduce the %swelling of the obtained hydrolyzed *M. indica* fruit starch. The smaller starch fragments may not swell as efficiently as partially hydrolyzed starch for shorter chains have lower ability to entangle and entrap the water molecules resulting in a less cohesive structure that swells less. Moreover, the higher temperature may have provided extensive hydrolysis, which produces soluble fragments that do not swell expressively.

Considering the data on the input factors influences on the output responses, the criteria were chosen for the optimization of hydrolyzed starch as presented in Table 6. Since both time and temperature showed significant impact on all output responses, all parameters were accounted for in the optimization. The lower Hausner ratio and Carr index presented superior flowability and compressibility. Therefore, the goals were set as minimums. While higher %swelling indicates better disintegrating property, this factor was set to be maximized. The experimental design proposed an optimized *M. indica* fruit starch hydrolysis reaction using 3.8 h at a temperature of 56.4 °C. The desirability score obtained was 0.871, indicating an outstanding data quality for the prediction and optimization [41]. After confirming the predicted responses using the optimized hydrolysis condition, it was found that the confirmed results using the optimized condition were comparable to the predicted data (*p* > 0.05). This assured that the prediction and optimization was reliable and could deliver predictable and reproducible outcomes.

### 3.3. Optimization of Pre-Gelatinized M. indica Fruit Starch

The optimization of pre-gelatinized *M. indica* fruit starch was also carried out using a computer-assisted approach in a microwave synthesizer. The rection time and temperature were the key parameters varied for the optimized pre-gelatinized starch. A 3-level factorial response–surface design produced 14 experimental runs, as detailed in Table 6. The reaction time (A) was between 1–5 min, whilst the reaction temperatures (B) were 100–150 °C. Following each pre-gelatinization, the obtained starch mixtures were slurry and viscous with a daker yellowish-orange color. This three-level factorial design and response surface methodology (RSM) was employed with a regression equation to identify the relationship between the independent and dependent variables. The analysis was conducted with a confidence interval of 95% and a *p*-value of 0.05.

Table 7 depicts the generated experiments and the results obtained for each investigated response. It was found that the flowability, presented by Hausner ratio, ranged from 1.05–1.25. This suggested that the flowability of the pregelatinized starch was fair to excellent which is distinct from the hydrolyzed starch. This could be because the pre-gelatinization process modified the starch into more uniform sizes and produced more spherical particles, which improves flowability through the reduction of interparticle friction and interlocking of irregular shapes. Carr indices also presented that the compressibility of the pregelatinized starch was superior to the hydrolyzed *M. indica* fruit starch. The range of the index was from 4.76–15, which was excellent to good compressibility. This could also owe to the improved particle size homogeneity and the spherical shape as well as the packing density of the modified *M. indica* fruit starch [37]. Lastly, the %swelling showed to improve drastically and ranged between 1200–1800%. These findings proposed the potential of the modified starch to act as a disintegrant in a tablet and presumably improve overall tablet performances [42]. Thus, other forms of hydrolyzed starch can be created using the relationship between time, temperature, and the responses.

The ANOVA and multiple regression analysis in Table 8 illustrated that the quadratic model was suitable for the prediction of Hausner ratio, Carr index, and %swelling with the model *p*-values of less than 0.05, lack-of-fit *p*-value greater than 0.05, and high R^2^ values that presented the suitability and accuracy of the mathematical model fitted for the prediction of the output responses. The coded equation from the multiple regression model proved that the time and temperature used in the reaction showed a negative trend towards the flowability and compressibility of the obtained starch, while only the temperature showed negative influence on %swelling. The model also suggested that the interaction of reaction time and temperature (AB) and the exponential relation of reaction time (A^2^) significantly impacted the %swelling of the modified starch (*p* < 0.05). Thus, the coded equations were able to predict the output responses with the given input factors from the relationships.

**Figure 2:**
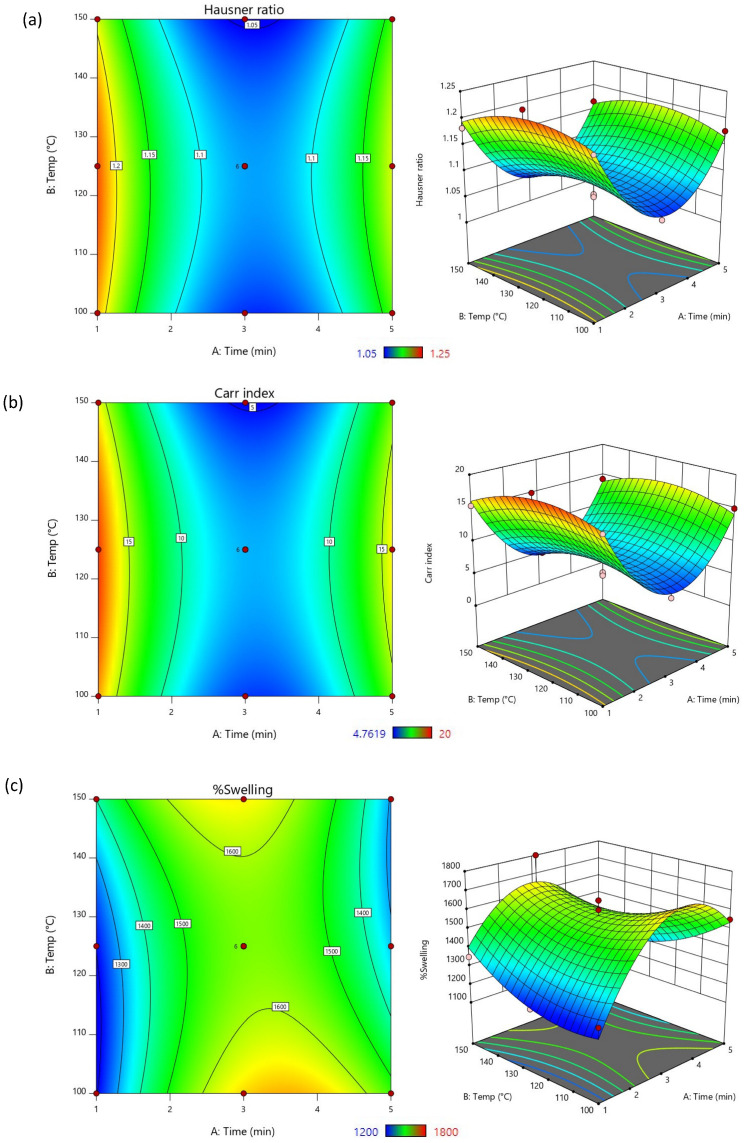
The contour plots and response surface graphs represent the effects of pre-gelatinization time and temperature on (**a**) Hausner ratio, (**b**) Carr index, and (**c**) %swelling.

The computer software also presented the relationships of the pre-gelatinized time and temperature with each of the output responses in 2D contour and 3D surface plot. As illustrated in Figure 2a,b, the Hausner ratio and Carr index decreased when the reaction time increased (presented in the blue region). On the other hand, these parameters positively impacted on the %swelling. When the interaction of reaction time and temperature were increased, the %swelling also increased (presented in the green area). However, the influences affected in an opposite direction when the conditions reached a certain point. It has been reported that overly processed starch can become too cohesive, sticky, and alter the particle shape irregularly, leading to poor flow properties and compressibility [43,44]. Additionally, the excessively processed starch via gelatinization may cause starch granules to lose their structural integrity, reducing their ability to swell effectively. Moreover, excessive swollen starch could cause the swelling molecules to collapse, diminishing their ability to swell further when re-hydrated [45]. The findings suggested that proper control of pre-gelatinization conditions is essential to optimize the functional properties of starch ensuring the desired balance of flowability, compressibility, and swelling.

The criteria to select the optimized condition for *M. indica* fruit starch pre-gelatinization was chosen using a similar rationale to the hydrolysis rection. That is, the Hausner ratio and Carr index were selected to be minimized, and the %swelling was set to be maximized in order to bring out the modified pre-gelatinized starch with excellent flow and compressibility as well as disintegrating property. The optimized condition for *M. indica* fruit starch pre-gelatinization using microwave synthesizer was at 150 °C for 3 min. As exemplified in Table 9, the obtained desirability of the data prediction was 0.767 which was considered acceptable [46]. The confirmation results of all output responses were similar to the predicted mean values assuring a successful optimization, and the relationship was amongst the parameters were established for the prediction of the results.

### 3.4. Characterizations of the Optimized Modified M. indica Fruit Starch

#### 3.4.1. Attenuated Total Reflection Fourier-Transformed Infrared Spectroscopy

**Figure 3:**
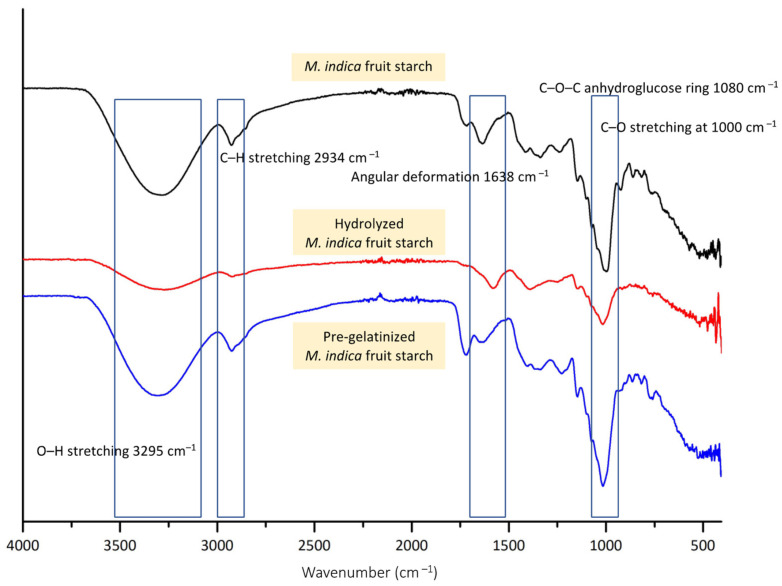
ATR-FTIR spectra of *M. indica* fruit starch hydrolyzed *M. indica* fruit starch and pre-gelatinized *M. indica* fruit starch.

The structural changes of modified *M. indica* fruit starch was examined using ATR-FTIR analysis. As shown in Figure 3, all starch formulations presented similar spectrums, with O–H stretching at 3295 cm^−1^, C–H stretching at 2934 cm^−1^, and C–O–C anhydroglucose ring at 1080 cm^−1^. Additionally, the C–O stretching was presented at 1000 cm^−1^ which belongs to the glycosidic links in the amylose and amylopectin structure. The spectra of unmodified *M. indica* fruit starch and pre-gelatinized starch were similar with the presence of the angular deformation of hydroxyl groups in glucose and residual water at 1638 cm^−1^. No significant changes were found in the pre-gelatinized starch spectrum for the reaction mainly affect the orderliness of the structure upon gelatinization leading to granular swelling, crystallite melting, loss of birefringence, viscosity enhancement, and improved solubilization. However, the hydrolysis reaction caused the breaking of glycosidic bonds at both α-1,4-glycosidic bonds in the amylose structure and the branched α-1,6-glycosidic bonds in the amylopectin chain [47]. The reaction could result in shorter chains and a reduction in molecular weight, especially amylose chains, which are more readily hydrolyzed than amylopectin, creating changes in the overall structure of starch. This is reflected in the spectrum of hydrolyzed *M. indica* fruit starch by reduced intensities of the bands, especially the O–H stretching possibly due to the reduced intra-molecular H-bond of the short chain starch. Additionally, the C–O stretching at 1000 cm^−1^ was altered indicating the breaking of glycosidic bonds. This complied with the findings on hydrolyzed cassava starch where the intensities were found to be less defined [48]. Therefore, the analyses assured that the reactions caused the structural changes among the modified starch.

#### 3.4.2. Differential Scanning Calorimetry

**Figure 4:**
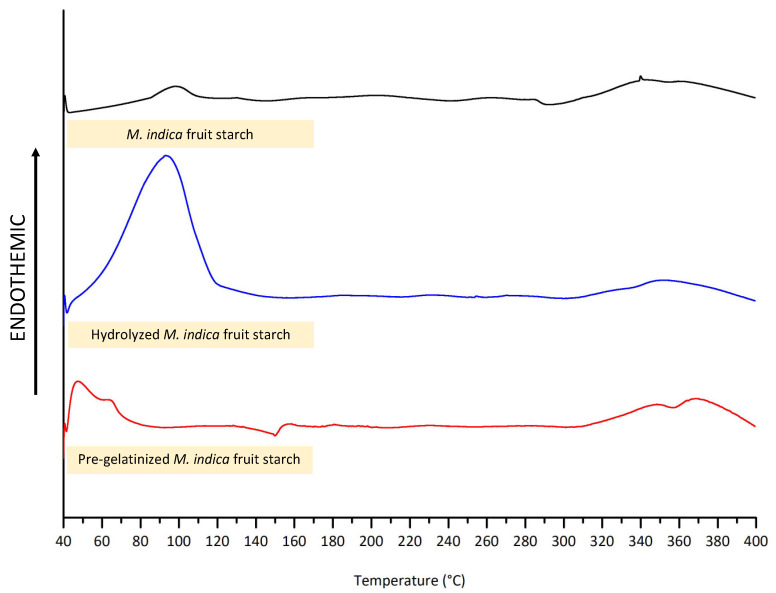
The DSC thermogram of intact and modified *M. indica* fruit starch.

The structural characteristics of the starch were also characterized using DSC. As depicted in Figure 4, the thermogram showed endothermic peaks at 98 °C for the unmodified starch showing the transition, which is the gelatinization of *M. indica* fruit starch, whereas the hydrolyzed starch showed relatively less gelatinization temperature at 93 °C possibly due to the shortened chain length. Interestingly, the gelatinization peak is absent in the DSC curve at the temperature found in for the intact starch. This could be because the pre-gelatinized *M. indica* fruit starch has already undergone gelatinization, with its crystalline regions completely disrupted during the pre-gelatinization process [49]. Moreover, the pre-gelatinized starch exhibited broaden and less defined endothermic transitions at 47 °C and 64 °C corresponding to the further relaxation or reorganization of the amorphous regions [50].

### 3.5. Modified Starch Performances

The performances of the optimized modified starch were compared to common conventional tableting excipients, which were MCC PH102 as the diluent, SSG as the super disintegrant, and PVP K30 as the binder. All powders were sieved through mesh No. 100 to control the particle size used in the experiments to allow comparable information. The flowability and compressibility of the optimized modified *M. indica* fruit starch and the excipients are presented in Table 10. The flowability and compressibility of MCC PH102 was considered passable, SSG was poor, which was in accordance with the literature’s reports [51,52]. MCC PH102 exhibited poor flow properties due to its hygroscopic nature, electrostatic charges, cohesive properties, and low bulk density. Specifically, hygroscopicity makes it readily absorb moisture from the environment causing the particles to stick together, forming agglomerates that hinder flow [53]. The findings suggested that the unmodified *M. indica* fruit starch may not be suitable to be used as direct compression tableting excipient as the flow and compressibility were considered poor. Moreover, the starch was not able to swell making it unfitting as disintegrant. Thus, the starch may be promising when other excipients and an appropriate tableting method, e.g., granulation, were involved [54]. The hydrolyzed *M. indica* fruit starch presented a significant improvement in its performances. The flowability was considered excellent with good compressibility. The %swelling was improved from MCC PH102 and the unmodified starch yet was approximately 0.31-fold as efficient as the super disintegrant (SSG). The hydrolyzed starch exhibited excellent flowability and good compressibility, primarily due to the breakdown of glycosidic bonds during hydrolysis. This process results in shorter chains of amylose and amylopectin, which enhance particle uniformity and reduce the tendency of particles to form agglomerates [55]. The improved solubility and dispersibility of hydrolyzed starch contribute to its potential as a direct compression diluent and even as a disintegrant in tablet formulations [56]. Surprisingly, the pre-gelatinized *M. indica* fruit starch presented a drastic change in the starch characteristics. The Hausner ratio and Carr index were in an excellent range, and the %swelling was greatly improved 3.95-fold and 1.24-fold compared to unmodified starch and SSG, respectively. This showed great potential of the pre-gelatinized *M. indica* fruit starch to be used as substituents for conventional pharmaceutical tableting excipients. The characteristics of this form of modified starch presented superior flowability and Carr index compared to MCC PH102 and empowered swelling property compared to SSG. Pre-gelatinization alters the crystalline structure of starch, increasing its amorphous content Pre-gelatinization alters the crystalline structure of starch [57]. This improves the starch’s surface area and reduces its crystallinity, enabling better particle cohesion and flowability [55]. The Hausner ratio and Carr index of the pre-gelatinized starch fell within excellent ranges, indicating minimal particle aggregation and better powder handling properties. Furthermore, the swelling capacity of the pre-gelatinized starch improved as it allows the starch to absorb more water and expand, facilitating faster tablet break-up in aqueous environments which is crucial for disintegration. The modification of *M. indica* fruit starch presented to be a promising approach to enhance the properties of the naturally derived starch. Formerly, pre-gelatinized tapioca starch exhibited good disintegration and dissolution effect when added to direct compressed tablet [14]. Furthermore, cassava starch was found to be a promising diluent, but cassava starch was not suitable to be used as a diluent in direct tablet compression due to it spoor compressibility and flow properties. Additional processes and excipients were required to improve the characteristics [15]. Herein, the optimized hydrolyzed and pre-gelatinized *M. indica* fruit starches were promising candidates to be used as tableting excipients and may be able to replace conventional inactive ingredients.

When the extracted and modified starch was used as a substituent for PVP K30 in direct compressed tablet, it was found that all tablets showed passable friability when tested under pharmacopeial condition with less than 1% weight lost. The tablet hardness is another important parameter indicating the function of the binder [58,59]. It was found that the unmodified and pre-gelatinized *M. indica* fruit starch, when used as binder, showed comparable hardness with PVP K30. Nevertheless, the hydrolyzed starch presented lower tablet hardness compared to PVP K30 due to reduced binding capacity. The shortening of amylose and amylopectin chains during hydrolysis leads to decreased molecular weight, resulting in fewer hydrogen bonds and weaker mechanical interlocking between particles [60]. This lower cohesion challenges its use as a binder, suggesting the need for a higher concentration or combination with other excipients to compensate for reduced binding forces [61]. Direct compression requires excipients with strong binding properties. Hydrolyzed starch having reduced binding capacity can be a challenge in tablet formulation that the concentration of hydrolyzed starch should be increased compared to using PVP K30 or used in combination. Thus, the shorter chain could be beneficial in wet granulation since hydrolyzed starch has improved solubility and dispersibility, which facilitates uniform granule formation with better flow and compressibility [62].

## 4. Conclusions

This study successfully demonstrated the potential of modified *M. indica* (mango) fruit starch as a substituent for conventional pharmaceutical tableting excipients. The application of acid hydrolysis and microwave-aided pre-gelatinization significantly enhanced the starch’s functional properties, including flowability, compressibility, and swelling capacity. The use of computer software-assisted optimization provided a robust framework for fine-tuning these modifications, leading to optimized starch with desirable characteristics for tablet formulation. The modified mango fruit starch exhibited comparable or superior performance to conventional excipients such as MCC PH102, SSG, and PVP K30, especially the pre-gelatinized starch. This research highlights the viability of using mango-derived starch as a sustainable, biocompatible, and cost-effective alternative in pharmaceutical applications. Modified *M. indica* fruit starch presented promising and favorable physicochemical properties. DOE can simultaneously optimize multiple response variables allowing precise tailoring of starch modification to achieve specific functional properties required for different pharmaceutical applications. The findings pave the way for further exploration of naturally derived excipients, promoting greener production processes and contributing to the circular economy by valorizing agricultural by-products. Further investigations may focus on the microscopic characteristics of the *M. indica* fruit starch and the evaluations on physical, chemical, and biological stabilities for natural compounds may prone to certain factors influencing changes in their properties.

## Figures and Tables

**Table 1:** The coded and actual range of each independent variable for the optimization of *M. indica* starch hydrolysis.

Factor	Name	Units	Type	Min	Max	Coded Low	Coded High
A	Time	h	Numeric	1.0000	6.00	−1 ↔ 1.00	+1 ↔ 6.00
B	Temperature	°C	Numeric	40.00	60.00	−1 ↔ 40.00	+1 ↔ 60.00

**Table 2:** The coded and actual range of each independent variable for the optimization of *M. indica* starch pre-gelatinization.

Factor	Name	Units	Type	Min	Max	Coded Low	Coded High
A	Time	min	Numeric	1.0000	5.00	−1 ↔ 1.00	+1 ↔ 5.00
B	Temperature	°C	Numeric	100.00	150.00	−1 ↔ 100.00	+1 ↔ 150.00

**Table 3:** Modified mango starch formulation as a replacement of binder. ^#^ Hydrolyzed *M. indica* fruit starch, pre-gelatinized *M. indica* fruit starch, or PVP K30.

Ingredients	%wt
Acetaminophen (325 mg)	73.86
Microcrystalline cellulose	20
Sodium starch glycolate	4
Binder ^#^	1
Magnesium stearate	1.14
Total	100

**Table 4:** Experimental runs generated from 3-level factorial response-surface design for hydrolysis optimization of *M. indica* fruit starch using DesignExpert^®^ software.

Run	Factor A	Factor B	Response (Y_1_)	Response (Y_2_)	Response (Y_3_)
	Time (h)	Temperature (°C)	Hausner Ratio	Carr Index	%Swelling
1	6	50	2	50	350
2	3.5	40	1.33	25	320
3	6	60	1.33	15	280
4	3.5	50	1.25	20	350
5	3.5	50	1.25	20	340
6	3.5	50	1.33	25	360
7	3.5	50	1.33	25	350
8	3.5	60	1	10	300
9	6	40	2	50	300
10	3.5	50	1.11	10	350
11	1	60	1.45	25	250
12	3.5	50	1.25	20	350
13	1	50	2	50	300
14	1	40	2	50	300

**Table 5:** Summary of the ANOVA and multiple regression analysis equations of the *M. indica* fruit starch hydrolysis optimization.

		Fit Summary			
	Model	*p*-Value	Lack of Fit	R^2^	Recommendation
Hausner ratio	Quadratic	<0.0001	0.1262	0.9091	Suggested
Carr index	Quadratic	0.0009	0.2123	0.8126	Suggested
%swelling	Quadratic	<0.0001	0.0826	0.9233	Suggested
Multiple regression model (Coded equation)					
Hausner ratio = 1.08 − 0.0205A − 0.0064B + 0.0047AB + 0.1310A^2^ − 0.0296B^2^ Carr index = 7.47 − 1.42A − 0.45B + 0.32AB + 10.22A^2^ − 2.28B^2^ %swelling = 350.29 + 13.33A − 15.00B + 7.50AB − 26.18A^2^ − 41.18B^2^					

**Table 6:** The criteria for the optimization and the confirmation of the hydrolyzed *M. indica* fruit starch.

Name	Goal	Solution	Desirability	Confirmation	*p*-Value
A: Time	is in range	3.802	0.871		
B: Temperature	is in range	56.40			
Hausner ratio	minimize	1.06 ± 0.11		1.09 ± 0.06	0.3587
Carr index	minimize	10.92 ± 6.55		11.38 ± 0.64	0.9140
%swelling	maximize	328.23 ± 9.27		336.67 ± 20.82	0.5564

**Table 7:** Experimental runs generated from 3-level factorial response-surface design for pre-gelatinization optimization of *M. indica* fruit starch using DesignExpert^®^ software and the output responses.

Run	Factor A	Factor B	Response 1	Response 2	Response 3
	Time (min)	Temperature (°C)	Hausner Ratio	Carr Index	%Swelling
1	3	150	1.136	12	1800
2	5	125	1.11	10	1400
3	1	150	1.05	5	1350
4	3	125	1.05	4.76	1500
5	3	125	1.18	15	1600
6	5	150	1.16	13.64	1200
7	3	125	1.05	4.76	1500
8	1	125	1.11	10	1200
9	1	100	1.25	20	1250
10	3	100	1.06	5.26	1600
11	5	100	1.13	11.11	1550
12	3	125	1.052	5	1500
13	3	125	1.052	5	1600
14	3	125	1.18	15	1650

**Table 8:** The summary of ANOVA and multiple regression analysis for the optimization of pre-gelatinized *M. indica* fruit starch.

		Fit Summary			
	Model	*p*-Value	Lack of Fit	R^2^	Recommendation
Hausner ratio	Quadratic	0.0002	0.8422	0.8237	Suggested
Carr index	Quadratic	0.0003	0.9060	0.8017	Suggested
%swelling	Quadratic	0.0018	0.0996	0.7243	Suggested
Multiple regression model (Coded equation)					
Hausner ratio = 1.11 − 0.003A − 0.014B + 0.058AB Carr index = 9.75 − 0.04A − 0.96B + 4.38AB %swelling = 1570.59 + 58.33A − 8.33B − 112.5AB − 307.35A^2^ + 92.65B^2^					

**Table 9:** The criteria for the optimization of pre-gelatinized *M. indica* fruit starch and the confirmation of the predicted responses.

Name	Goal	Solution	Desirability	Data Mean	*p*-Value
A: Time	is in range	3	0.767		
B: Temperature	is in range	150			
Hausner ratio	minimize	1.08 ± 0.03		1.03 ± 0.06	0.2663
Carr index	minimize	7.47 ± 2.37		4.27 ± 0.25	0.1428
%swelling	maximize	1360.71 ± 280.23		1326.67 ± 25.17	0.8531

**Table 10:** Hausner ratio, Carr index, and %swelling of the conventional excipient compared to *M. indica* fruit starch and tablet performances when starch was used as PVP K30 substitute (Significant difference, *p* < 0.05, compared to ^#^ MCC PH102, ^&^ SGG, ^$^ PVP K30, * unmodified *M. indica* fruit starch).

Excipient	Hauner Ratio		Carr Index		%Swelling
MCC PH102	1.28 ± 0.02	Passable	21.83 ± 1.04	Passable	0.00 ± 0.00
SSG	1.35 ± 0.01	Poor	26.00 ± 0.50	Poor	1066.67 ± 28.87
PVP K30	1.46 ± 0.01	Very Poor	31.67 ± 0.58	Poor	Soluble
Unmodified *M. indica* fruit starch	1.38 ± 0.01 ^#$^	Poor	27.50 ± 0.50 ^#$^	Poor	0.00 ± 0.00 ^&^
Hydrolyzed *M. indica* fruit starch	1.09 ± 0.06 ^#&$^*	Excellent	11.38 ± 0.64 ^#&$^*	Good	336.67 ± 20.82 ^&^*
Pre-gelatinized *M. indica* fruit starch	1.03 ± 0.06 ^#&$^*	Excellent	4.27 ± 0.25 ^#&$^*	Excellent	1326.67 ± 25.17 ^&^*
**Tablet Performances**					
Binder		Hardness		Friability	
PVP K30 tablet		8.79 ± 1.32		PASS	
Unmodified *M. indica* fruit starch		8.68 ± 0.57		PASS	
Hydrolyzed *M. indica* fruit starch		6.23 ± 1.16 ^@^		PASS	
Pre-gelatinized *M. indica* fruit starch		8.81 ± 1.32		PASS	

^@^ Significant difference compared to PVP K30 (*p* < 0.05).

## Data Availability

The original contributions presented in the study are included in the article, further inquiries can be directed to the corresponding author.
